# DNA methylation status of *REIC/Dkk*-*3* gene in human malignancies

**DOI:** 10.1007/s00432-012-1158-6

**Published:** 2012-01-25

**Authors:** Tatsuro Hayashi, Hiroaki Asano, Shinichi Toyooka, Kazunori Tsukuda, Junichi Soh, Tadahiko Shien, Naruto Taira, Yuho Maki, Norimitsu Tanaka, Hiroyoshi Doihara, Yasutomo Nasu, Nam-ho Huh, Shinichiro Miyoshi

**Affiliations:** 1 Department of Cancer and Thoracic Surgery, Graduate School of Medicine, Dentistry and Pharmaceutical Sciences, Okayama University, 2-5-1 Shikata-cho, Okayama, 700-8558 Japan; 2 Department of Urology, Graduate School of Medicine, Dentistry and Pharmaceutical Sciences, Okayama University, Okayama, Japan; 3 Department of Cell Biology, Graduate School of Medicine, Dentistry and Pharmaceutical Sciences, Okayama University, Okayama, Japan

**Keywords:** DNA methylation, *REIC/Dkk*-*3*, Breast cancer, Lung cancer, Mesothelioma

## Abstract

**Purpose:**

The *REIC (reduced expression in immortalized cells)/Dkk*-*3* is down-regulated in various cancers and considered to be a tumor suppressor gene. *REIC/Dkk*-*3* mRNA has two isoforms (type-a,b). *REIC* type-a mRNA has shown to be a major transcript in various cancer cells, and its promoter activity was much stronger than that of type-b. In this study, we examined the methylation status of *REIC/Dkk*-*3* type-a in a broad range of human malignancies.

**Methods:**

We examined *REIC/Dkk*-*3* type-a methylation in breast cancers, non-small-cell lung cancers, gastric cancers, colorectal cancers, and malignant pleural mesotheliomas using a quantitative combined bisulfite restriction analysis assay and bisulfate sequencing. *REIC/Dkk*-*3* type-a and type-b expression was examined using reverse transcriptional PCR. The relationships between the methylation and clinicopathological factors were analyzed.

**Results:**

The rate of *REIC/Dkk*-*3* type-a methylation ranged from 26.2 to 50.0% in the various primary tumors that were examined. *REIC/Dkk*-*3* type-a methylation in breast cancer cells was significantly heavier than that in the other cell lines that we tested. *REIC/Dkk*-*3* type-a methylation was inversely correlated with *REIC/Dkk*-*3* type-a expression. There was a correlation between *REIC/Dkk*-*3* type-a and type-b mRNA expression. *REIC/Dkk*-*3* type-a expression was restored in MDA-MB-231 cells using 5-aza-2′-deoxycytidine treatment. We found that estrogen receptor–positive breast cancers were significantly more common among the methylated group than among the non-methylated group.

**Conclusions:**

*REIC/Dkk*-*3* type-a methylation was frequently detected in a broad range of cancers and appeared to play a key role in silencing *REIC/Dkk*-*3* type-a expression in these malignancies.

## Introduction

Accumulating evidence suggests that tumor progression is governed not only by genetic changes intrinsic to cancer cells but also by epigenetic changes. In cancer epigenetics, aberrant CpG methylation in the promoter region is a key mechanism for gene inactivation, resulting in tumorigenesis in human malignancies (Toyooka and Shimizu 2004).

The *REIC (reduced expression in immortalized cells)/Dkk*-*3(Dickkopf*-*3)* cDNA, which was expressed in human normal cells and was down-regulated in human immortalized cells and human tumor-derived cells, was identified using a representative difference analysis system (Tsuji et al. 2000). The amino acid sequence revealed that the *REIC* gene product was human *Dkk*-*3*, one of the Dkk family members. The Dkk family of secreted proteins consists of four members, which share two conserved cysteine-rich domains (Glinka et al. 1998; Krupnik et al. 1999). Dkk-1, the best-characterized member of the Dkk family, functions as a Wnt antagonist or agonist by binding to and inhibiting or activating the Wnt coreceptor LRP6 (Bafico et al. 2001). Unlike Dkk-1, Dkk-2, and Dkk-4, however, REIC/Dkk-3 was recently shown to inhibit TCF-4 receptor activity in lung cancer cells (Yue et al. 2008). TCF-4 activates c-Myc and cyclin D1 through the Wnt/beta-catenin pathway and promotes tumor invasion and metastasis. Because *REIC/Dkk*-*3* is down-regulated in a variety of malignancies and the overexpression of *REIC/Dkk*-*3* suppresses cell growth, *REIC/Dkk*-*3* has been proposed to act as a tumor suppressor (Tsuji et al. 2001; Kurose et al. 2004). Hypermethylation and the down-regulation of *REIC/Dkk*-*3* were observed in a variety of malignancies including non-small-cell lung cancers (NSCLCs) (Kobayashi et al. 2002; Licchesi et al. 2008), gastrointestinal cancers (Maehata et al. 2008), renal clear cell carcinoma (Kurose et al. 2004), acute lymphoblastic leukemia (Roman-Gomez et al. 2004) and osteosarcomas (Hoang et al. 2004). We previously showed the therapeutic effect of REIC/Dkk-3 in prostate cancers (Abarzua et al. 2005; Edamura et al. 2007) and malignant pleural mesothelioma (MPM) (Kashiwakura et al. 2008). In addition, tumor suppression by REIC/Dkk-3 has also been confirmed in other malignant tumors (Hsieh et al. 2004; Hoang et al. 2004).

*REIC/Dkk*-*3* mRNA has two isoforms (type-a,b; GenBank accession AB057804). Many papers have described the methylation status in the promoter of *REIC/Dkk*-*3* type-b (Licchesi et al. 2008; Maehata et al. 2008; Veeck et al. 2009). However, the promoter of *REIC/Dkk*-*3* type-a also seems to be important, since Kobayashi et al. (2002) (the group that first identified the *REIC/Dkk*-*3* in immortalized cells) have demonstrated that the promoter activity of *REIC/Dkk*-*3* type-a (major promoter) had an approximately 26-fold stronger effect than that of *REIC/Dkk*-*3* type-b (minor promoter) in a luciferase assay, and the major transcript was *REIC/Dkk*-*3* type-a in various cancer cells they tested. They suggested that hypermethylation of the major promoter (type-a) was a major mechanism for the down-regulation of *REIC* expression. They also suggested that the methylation of the minor promoter (type-b) was accompanied with that of major promoter (type-a) in most cases except four lung cancer cells that they tested. Regardless, those four lung cancer cells had type-b hypermethylation, *REIC/Dkk*-*3* type-b expression was detected in those four lung cancer cells. So they discussed the possibility that minor promoter (type-b) was utilized for the expression in a tissue-specific manner, as seen in dual promoter of APC gene.

In this study, we examined the DNA methylation of *REIC/Dkk*-*3* type-a in various kinds of cancers by quantitative combined bisulfite restriction analysis (qCOBRA) and investigated the correlation between the *REIC/Dkk*-*3* type-a methylation and *REIC/Dkk*-*3* type-a expression. The qCOBRA assay can provide more reliable results because the conventional methylation-sensitive restriction enzyme assay that Kobayashi et al. (2002) performed was recently known to be prone to false-positive results due to spurious incomplete digestion (Xiong and Laird 1997). We also analyzed the correlation between *REIC/Dkk*-*3* type-a and type-b expression in various cancer cell lines. Furthermore, we examined the correlation between *REIC/Dkk*-*3* type-a methylation and the clinicopathological features of primary tumors.

## Materials and methods

### Clinical samples and cell culture

Surgically resected specimens of 37 primary breast cancers, 42 primary NSCLCs, 21 primary gastric cancers, 20 primary colon cancers, and 7 MPMs were obtained from Okayama University Hospital (Okayama, Japan), 6 MPMs were obtained from Okayama Rousai Hospital (Okayama, Japan), 5 MPMs were obtained from National Sanyo Hospital (Yamaguchi, Japan), and 27 MPMs were obtained from Karmanos Cancer Center (MI). Ten corresponding non-malignant breast tissues and 10 non-malignant lung tissues were also examined. All tissues were frozen with the liquid nitrogen immediately after surgery and were stored at −80°C until extraction of DNA. Institutional Review Board permission and informed consent were obtained for all cases.

Seven breast cancer cell lines (HCC70, HCC1599, HCC1806, MDA-MB-231, MDA-MB-361, MCF7, and ZR75-1), 11 lung cancer cell lines (NCI-H23, NCI-H44, NCI-H125, NCI-H157, NCI-H1299, NCI-H1819, NCI-H1963, NCI-H1975, NCI-H2009, NCI-H358, and A549), 4 MPM cell lines (NCI-H2052, NCI-H2373, NCI-H2452, and NCI-H290), and 6 prostate cancer cell lines (PC3, LNCap-FGC, Du145, Caki-1, Caki-2, and KPK) were examined in this study. MCF7, ZR-75-1, MDA-MB-231, and MDA-MB-361 were obtained from Cell Resource Center for Biomedical Research Institute of Development Aging and Cancer Tohoku University (Miyagi, Japan). Seven cell lines (HCC70, HCC1599, HCC1806, H2052, H2373, H290, and H2452) were kind gifts from Adi F. Gazdar (Department of Pathology, University of Texas Southwestern Medical Center, Dallas, TX). Six cell lines (PC3, LNCap-FGC, Du145, Caki-1, Caki-2, and KPK) were kind gifts from the department of urology (Okayama University, Okayama, Japan). The other cell lines were obtained from American Type Culture Collection (Manassas, VA). The cells were maintained in RPMI-1640 medium (Sigma Chemical Co., Saint Louis, MO) supplemented with 10% FBS and were incubated in 5% CO_2_.

### DNA extraction and DNA methylation modification

Genomic DNA was extracted from the surgically resected frozen samples and cultured cells by digestion with SDS/proteinase K followed by phenol/chloroform (1:1) extraction and ethanol precipitation. Two micrograms of each DNA was treated with EZ DNA Methylation Kit (ZYMO RESEARCH, Orange, CA), following the manufacturer’s instructions, and was stored at −20°C until use.

### Quantitative COBRA assay

**Fig. 1:**
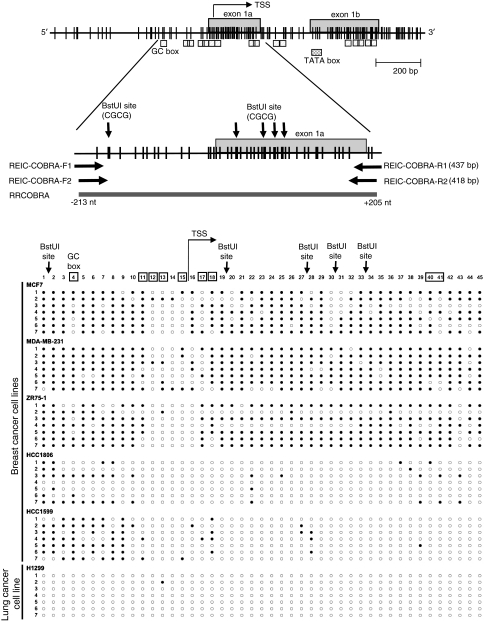
Map of the 5′-flanking region of *REIC/Dkk*-*3* and the bisulfite genomic DNA sequence. *Upper* figure demonstrates the promoter region of *REIC/Dkk*-*3*. *Gray bars* indicate exons, and the *bent arrow* indicates the transcription start site (TSS) (+1). *Thin vertical lines* on the *horizontal line* indicate the sites of CpG dinucleotides. *Arrow heads* indicate the restriction sites of BstUI. COBRA primer sets are indicated by *pairs of bold arrows*. *Dark gray bar* under the COBRA primer sets indicates RRCOBRA. Methylation status of individual subcloned DNA fragments of each cell line is shown *below*. Each *circle* represents a CpG dinucleotide in 5′-flanking region of *REIC/Dkk*-*3* (for −213 to +205 nt). The *numbers* at the *top* indicate the CpG dinucleotide in the RRCOBRA (from 5′ to 3′). These numbers correspond to those depicted in *upper* figures. *Open circle* represents non-methylated CpG dinucleotide. *Black circle* represents methylated CpG dinucleotide

Nested PCR was carried out using bisulfite-treated DNA followed by the restriction enzyme digestion. First-round touchdown PCR was performed under the following conditions: 95°C for 12 min, 40 cycles of 94°C for 45 s, annealing temperature between 58 and 56°C for 1 min, 72°C for 3 min, followed by final extension step at 72°C for 7 min in a 25-μl reaction mixture containing 67 mM Tris–HCl (pH 8.8), 16.6 mM (NH_4_)_2_SO_4_, 6.7 mm MgCl_2_, 10 mM β-mercaptoethanol, 1.25 mM of each deoxynucleotide triphosphate (dNTP) mixture, 0.5 μM of each primer, 0.5 unit of HotStar Taq DNA Polymerase (Qiagen, Valencia, CA), and 100 ng of bisulfite-treated DNA. Second-round touchdown PCR was performed using 0.4 μl of the first-round PCR products as a template under same condition, but 47 cycles. Universal methylated DNA and universal unmethylated DNA were used for positive control and negative control, respectively. The location of the CpG dinucleotides in the exon1 and in the 5′-flanking region of *REIC/Dkk*-*3* is shown in Fig. 1. Primers were designed using Primer Express software ver.1.0 in the promoter region of *REIC/Dkk*-*3* type-a. Primers for the first-round PCR were *REIC*-COBRA-F1 5′-TGGGTTGTTGTAAGTTTGAAGGT-3′ and *REIC*-COBRA-R1 5′-CTCACCCACCCCRACTAAAC-3′. Primers for the second-round PCR were as follows: *REIC*-COBRA-F2 5′-TGAAGGTTAGATAAGAYGGGTTTAGG-3′ and *REIC*-COBRA-R2 5′-ACCCACCCCRACTAAACCRAAT-3′. These primers were designed to ensure amplification of both methylated and unmethylated forms. Two microliters of second PCR products were digested with 3 units of BstUI (whose restriction site is CGCG) for the restriction fragment length polymorphism analysis. The amplicon of second PCR was named RRCOBRA (Region for REIC-COBRA), and the 5 restriction sites of BstUI are shown in Fig. 1. The digested PCR products were visualized on 3% agarose gels stained with ethidium bromide. The percentages of digested band were analyzed by NIH ImageJ 1.37 V software (http://rsb.info.nih.gov/ij) as described previously (Xiong and Laird 1997). We performed linear regression analysis of qCOBRA with nested PCR using serial dilution to examine whether qCOBRA with nested PCR really reflected % methylation. We diluted unmethylated DNA amplicon with methylated amplicon to make serial dilution (% methylated DNA; 0, 10, 20, 30, 50, 70, 80, 90, 100%) and performed qCOBRA, as described above.

### Bisulfited DNA sequencing analysis

RRCOBRA was cloned into pCR2.1-TOPO Vector using TOPO TA cloning kit (Invitrogen Life Technologies, Carlsbad, CA) following manufacturer’s instructions. To determine the methylation status in the promoter lesion of *REIC/Dkk*-*3* gene, five breast cancer cell lines (MCF-7, MDA-MB-231, ZR75-1, HCC1806, and HCC1599) and a lung cancer cell line (H1299) were examined. Seven individual clones from each cell line were sequenced using the dGTP BigDye terminator v3.1 Cycle Sequencing Kit with the ABI PRISM 3100 Genetic Analyzer (Applied Biosystems, Foster City, CA).

### RNA extraction and reverse transcriptional (RT)-PCR

Total RNA was extracted from cultured cells using RNeasy Mini Kit (Qiagen, Valencia, CA) following the manufacturer’s instruction. Oligo(dT)-primed cDNA was synthesized using Super-Script II (Qiagen, Valencia, CA) with DNase treatment. RT-PCR was carried out in 20 μl of reaction mixture with 1xPCR buffer, 200 μM of dNTP, 0.3 μM of each primer, 0.5 units of HotStarTag DNA Polymerase, and 100 ng of cDNA. A touchdown PCR was performed for *REIC/Dkk*-*3* type-a and type-b under the following conditions: 95°C for 12 min, 35 cycles of 94°C for 30 s, annealing temperature between 62 and 58°C for 1 min, 72°C for 3 min, followed by final extension step at 72°C for 7 min. As an internal control, RT-PCR for *GAPDH* was carried out under the following conditions: 95°C for 12 min, 35 cycles of 94°C for 45 s, 55°C for 90 s, 72°C for 90 s, followed by final extension step at 72°C for 7 min. The primers for *REIC/Dkk*-*3* type-a expression were *REIC*(a)-F 5′-GGGAGCGAGCAGATCCAGT-3′ (exon1a) and *REIC*(a)-R 5′-TTTGTCCAGTCTGGTTGTTGGT-3′ (exon3). The primers for *REIC/Dkk*-*3* type-b expression were *REIC*(b)-F 5′-TGGGAGCTATTAGCGTAGAGGAT-3′ (exon1b) and *REIC*(b)-R 5′-CATTGTGATAGCTGGGAGGTAAG-3′ (exon3). The PCR products were visualized on 2% agarose gels stained with ethidium bromide. The bands were analyzed using NIH ImageJ 1.37 V software. The expression ratio in each cell line was defined as the ratio of particular sample when compared to those of H1299. To confirm the responsibility of DNA methylation for *REIC/Dkk*-*3* silencing, we treated heavily methylated cell lines (MDA-MB-231) with 5-aza-2′-deoxycytidine (5-Aza-CdR) at the concentration of 5 and 8 μM for 6 days with medium changes on days 1, 3, and 5. Treated and untreated cells from individual triplicate flasks were harvested on day 6 to determine the *REIC/Dkk*-*3* type-a expression using RT-PCR.

### ER, PgR, and HER2 status in primary breast cancers

Estrogen receptor (ER), progesterone receptor (PgR), and HER2 status in primary breast cancers were obtained from patient medical records. HER2 positive was defined as a score of 2+ and 3+ by immunohistochemistry.

### Statistical analyses

The frequencies of *REIC/Dkk*-*3* methylation between two groups were compared using the Fisher’s exact test or Mann–Whitney’s *U* test when appropriate. Probability value less than 0.05 was defined as being statistically significant. All data were analyzed by JMP9 for Windows (SAS Institute, Cary, NC).

## Results

### DNA methylation status in the promoter region of *REIC/Dkk*-*3* type-a

The results of bisulfite genomic DNA sequencing of RRCOBRA are shown in Fig. 1. Each CpG in the 5′-flanking region and in exon1a was heavily methylated in MCF7, MDA-MB-231, and ZR75-1. The CpGs in the 5′-flanking region of exon1a were lightly to moderately methylated, but the CpGs in exon1a were rarely methylated in HCC1806 and HCC1599. In contrast, most of the CpGs were rarely methylated in H1299.

**Fig. 2:**
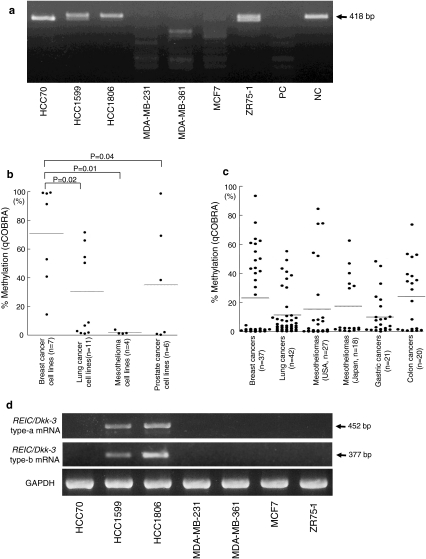
Quantitative COBRA assay. The results of COBRA assay for *REIC/Dkk*-*3* type-a in breast cancer cell lines were shown in **a**. Methylated alleles were fragmented with restriction enzyme modification, and unmethylated alleles were uncut. Percentages of digested band in cell lines (**b**) and primary tumors (**c**) were quantitated by NIH ImageJ 1.37 V software. The *horizontal bars* indicate the average in each group. The mRNA expression of *REIC/Dkk*-*3* type-a and type-b in breast cancer cell lines was shown in **d**. *PC* universal methylated DNA as positive control; *NC* universal unmethylated DNA as negative control

We performed the linear regression analysis using the nested qCOBRA and confirmed the quantitative capacity (data not shown). Representative examples of the COBRA assay in breast cancer cell lines are shown in Fig. 2a. The percentages of *REIC/Dkk*-*3* type-a methylation were calculated by qCOBRA in each cell line and primary tumor (Fig. 2b, c, respectively) and summarized in Table 1. We decided the samples with more than 10% of digested bands as methylation positive in this study. Aberrant methylation was detected in 7 of the 7 (100%) breast cancer cell lines, 16 of the 37 (43.2%) primary breast cancers, 5 of the 11 (45.4%) lung cancer cell lines, 11 of the 42 (26.2%) primary lung cancers, 0 of 4 (0%) MPM cell lines, 7 of the 27 (25.9%) USA primary MPMs, 7 of the 18 (38.9%) Japanese primary MPMs, 8 of the 21 (38.1%) primary gastric cancers, and 10 of the 20 (50.0%) primary colon cancers. *REIC/Dkk*-*3* methylation was not detected in 10 normal breast tissues and 10 normal lung tissues (data not shown). The methylation of *REIC/Dkk*-*3* type-a in the breast cancer cell lines was more frequent than that in the lung, MPM, and prostate cancer cell lines (*p* = 0.02, *p* = 0.01, and *p* = 0.04, respectively). However, no significant differences in methylation were observed among the primary breast, lung, MPMs, gastric, and colon cancers (Fig. 2c). The results of qCOBRA in five breast cancer cell lines and a lung cancer cell line (H1299) corresponded with the results of bisulfite sequencing.

**Table 1:** Rate of *REIC/Dkk3* methylation in each human cancer by quantitative COBRA assay

Organ	Number of *REIC/Dkk3* methylated sample (%)	
	Cell lines	Primary tumors
Breast cancer	7 of 7 (100%)	16 of 37 (43.2%)
Lung cancer	5 of 11 (45.4%)	11 of 42 (26.2%)
Malignant pleural mesothelioma		
USA	0 of 4 (0%)	7 of 27 (25.9%)
Japanese	ND	7 of 18 (38.9%)
Gastric cancer	ND	8 of 21 (38.1%)
Colon cancer	ND	10 of 20 (50.0%)
Prostate cancer	3 of 6 (50%)	ND

*ND* not determined

### *REIC/Dkk*-*3* mRNA expression in cell lines and correlation with qCOBRA assay

**Fig. 3:**
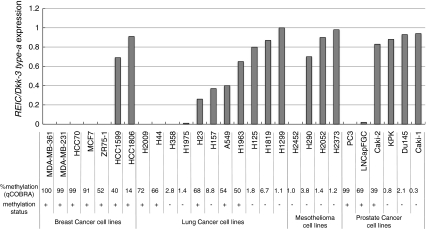
Relative expression of *REIC/Dkk*-*3* type-a mRNA and percentages of *REIC/Dkk*-*3* methylation in each cell line. *Columns* show the relative expression of *REIC/Dkk*-*3* type-a mRNA in each sample. The expression ratio was defined as the ratio of particular sample when compared to those of H1299. % methylation was calculated by qCOBRA assay

**Fig. 4:**
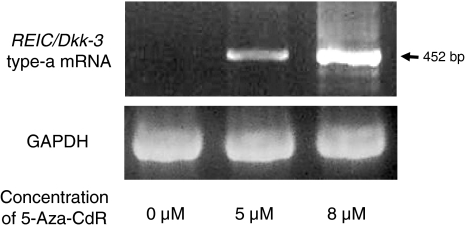
Restoration of *REIC/Dkk*-*3* type-a mRNA expression in MDA-MB-231 cells. The effect of 5-Aza-CdR on the restoration of *REIC/Dkk*-*3* type-a mRNA expressions in heavily methylated breast cancer cells (MDA-MB-231). *REIC/Dkk*-*3* type-a mRNA was detected by RT-PCR. GAPDH was used as an internal control

Representative example of RT-PCR for *REIC/Dkk*-*3* type-a and type-b in the breast cancer cell lines was shown in Fig. 2d. Expression of *REIC/Dkk*-*3* type-a was only detected in HCC1599 and HCC1806 cells, which rarely to moderately harbored *REIC/Dkk*-*3* methylation. Expression of *REIC/Dkk*-*3* type-b was also detected in HCC1599 and HCC1806 cells. The results of expression of *REIC/Dkk*-*3* type-a and type-b in all cell lines (*n* = 28) were summarized in Table 2. There was a correlation between the expression of *REIC/Dkk*-*3* type-a and type-b (*p* < 0.01). The relative expressions of *REIC/Dkk*-*3* type-a mRNA and the percentages of *REIC/Dkk*-*3* type-a methylation are shown in Fig. 3. The expression of *REIC/Dkk*-*3* type-a and *REIC/Dkk*-*3* type-a methylation was inversely correlated in the cell lines that were examined (*p* < 0.01). To confirm that methylation was responsible for the gene silencing, heavily methylated MDA-MB-231 cells were treated with 5-Aza-CdR. *REIC/Dkk*-*3* type-a mRNA expression was restored by the treatment of 5-Aza-CdR in a dose-dependent manner (Fig. 4).

**Table 2:** Expression of *REIC/Dkk*-*3* type-a and type-b in various cancer cells

Organ	mRNA expression	
	Type-a	Type-b
*Breast cancers*		
HCC70	−	−
HCC1599	+	+
HCC1806	+	+
MDA-MB-231	−	−
MDA-MB-361	−	−
MCF7	−	−
ZR75-1	−	−
*Lung cancers*		
H23	+	−
H44	−	−
H125	+	−
H157	+	+
H1299	+	+
H1819	+	+
H1963	+	+
H1975	−	+
H2009	−	−
H358	−	−
A549	+	+
*MPMs*		
H2052	+	+
H2373	+	+
H2452	−	−
H290	+	+
*Prostate cancers*		
Caki-1	+	+
Caki-2	+	+
Du145	+	+
KPK	+	+
LNCap-FGC	−	−
PC3	−	−

### *REIC/Dkk*-*3* methylation and clinicopathological correlation

We next examined the relationships between the *REIC/Dkk*-*3* methylation status and the clinicopathological factors described in Table 3. For the breast cancers, we observed that ER-positive cases were more common in the methylated group than in the non-methylated group (*p* = 0.03). No significant relationships between *REIC/Dkk*-*3* methylation and the other clinicopathological factors were observed.

**Table 3:** Clinicopathological factors and *REIC/Dkk*-*3* methylation in various primary cancers

Variables	Number of methylation-positive samples (%)
Total (*n* = 38)	16 (39)
*A. Primary breast cancers*	
Age	
<65 (*n* = 32)	14 (44)
≥65 (*n* = 6)	1 (17)
Histology	
Papillotubular (*n* = 8)	2 (25)
Solid-tubular (*n* = 11)	4 (36)
Scirrhous (*n* = 17)	9 (53)
Others (*n* = 2)	0
T categories	
1 (*n* = 11)	6 (55)
2 (*n* = 16)	5 (31)
3 (*n* = 3)	1 (33)
4 (*n* = 8)	3 (38)
N categories	
0 (*n* = 15)	6 (40)
1 (*n* = 23)	9 (39)
M categories	
0 (*n* = 36)	14 (23)
1 (*n* = 2)	1 (50)
Stage	
I (*n* = 9)	5 (56)
II (*n* = 15)	5 (33)
III (*n* = 12)	4 (33)
IV (*n* = 2)	1 (50)
Estrogen receptor*	
Positive (*n* = 19)	11 (58)
Negative (*n* = 18)	4 (22)
Progesterone receptor	
Positive (*n* = 18)	9 (50)
Negative (*n* = 15)	6 (40)
HER2 status	
Positive (*n* = 11)	3 (27)
Negative (*n* = 19)	10 (53)

Total (*n* = 41)	11 (27)
*B. Primary lung cancers*	
Age	
<65 (*n* = 17)	3 (18)
≥65 (*n* = 24)	8 (33)
Histology	
Adenocarcinoma (*n* = 27)	7 (26)
Squamous cell carcinoma (*n* = 14)	4 (29)
T categories	
1 (*n* = 27)	7 (26)
2 (*n* = 12)	4 (33)
3 (*n* = 0)	0
4 (*n* = 2)	0
N categories	
0 (*n* = 29)	8 (28)
1 (*n* = 10)	2 (20)
M categories	
0 (*n* = 39)	11 (28)
1 (*n* = 2)	0
Stage	
I (*n* = 28)	8 (29)
II (*n* = 4)	1 (25)
III (*n* = 7)	1 (14)
IV (*n* = 2)	0

Total (*n* = 21)	8 (38)
*C. Primary gastric cancers*	
Age	
<65 (*n* = 10)	5 (50)
65≤ (*n* = 11)	3 (27)
Histology	
Intestinal (*n* = 10)	5 (50)
Diffuse (*n* = 11)	3 (27)
T categories	
1 (*n* = 3)	1 (55)
2 (*n* = 8)	4 (50)
3 (*n* = 7)	2 (29)
4 (*n* = 3)	0
N categories	
0 (*n* = 7)	4 (57)
1 ≤ (*n* = 14)	4 (29)
Stage	
I (*n* = 6)	4 (67)
II (*n* = 3)	1 (33)
III (*n* = 5)	3 (60)
IV (*n* = 7)	0

Total (*n* = 20)	10 (50)
*D. Primary colon cancers*	
Age	
<65 (*n* = 9)	5 (56)
65≤ (*n* = 11)	5 (46)
Histology	
Well (*n* = 5)	1 (20)
Moderately (*n* = 11)	6 (55)
Poorly (*n* = 3)	2 (67)
Others (*n* = 1)	1 (100)
T categories	
1 (*n* = 2)	1 (50)
2 (*n* = 0)	0
3 (*n* = 14)	6 (43)
4 (*n* = 3)	3 (100)
N categories	
0 (*n* = 8)	4 (50)
1 ≤ (*n* = 12)	6 (50)
M categories	
0 (*n* = 12)	6 (50)
1 (*n* = 8)	4 (50)
Stage	
I (*n* = 3)	2 (67)
II (*n* = 4)	1 (25)
III (*n* = 5)	3 (60)
IV (*n* = 8)	4 (50)
Location	
Right (*n* = 8)	5 (63)
Left (*n* = 11)	4 (36)

* *p* < 0.05

## Discussion

In this study, we demonstrated that arbitrary CpG methylation in *REIC/Dkk*-*3* type-a promoter region was frequently observed in solid malignancies. Regarding qCOBRA in this study, we confirmed the accuracy of this assay by linear regression analysis because we performed nested PCR. We decided the samples with more than 10% of digested bands as methylation positive to maintain compatibility with conventional COBRA assay, as we could distinguish 10% of digested band on the agarose gel electrophoresis. Colella et al. (2003) also used a 10% threshold to declare methylation when qCOBRA was compared with pyrosequencing methylation analysis. So a 10% threshold seems to be reasonable criteria to distinguish methylation positive. We examined cell lines using qCOBRA assay, and the accuracy of the qCOBRA was also confirmed by bisulfate genomic DNA sequencing and linear regression analysis.

The *REIC/Dkk*-*3* type-a methylation, which was determined using qCOBRA assay, revealed to be inversely correlated with the *REIC/Dkk*-*3* type-a mRNA expression among the cell lines (*p* < 0.01), and the restoration of *REIC/Dkk*-*3* type-a expression by 5-Aza-CdR treatment was observed in a *REIC/Dkk*-*3* type-a methylated cell line. These results indicate that DNA methylation of *REIC/Dkk*-*3* type-a was responsible for silencing *REIC/Dkk*-*3* type-a expression. As reported previously, we confirmed that there was a correlation between *REIC/Dkk*-*3* type-a expression and type-b expression in the cell lines that we examined (*p* < 0.01). Discrepancy of the expression level between *REIC/Dkk*-*3* type-a and type-b was observed in lung cancer cell lines, although the expression of *REIC/Dkk*-*3* type-a completely corresponded with the expression of *REIC/Dkk*-*3* type-b in other cell lines, indicating that *REIC/Dkk*-*3* type-b might be utilized for the expression in a tissue-specific manner, as Kobayashi et al. (2002) described.

Among the cancers that were examined, *REIC/Dkk*-*3* type-a methylation was more frequently detected in breast cancer cell lines, although moderate methylation was also observed in other cancers. A previous report showed that the introduction of REIC/Dkk-3 into some breast cancer cells had an antitumor effect (Kawasaki et al. 2009). In addition, the introduction of REIC/Dkk-3 into cancer cells had a direct effect on the induction of apoptosis and an indirect effect on the activation of tumor immunity in NK cells through the up-regulation of IL-7 (Sakaguchi et al. 2009). Furthermore, REIC/Dkk-3 induces the differentiation of human CD14+ monocytes into a novel cell type, resembling immature dendritic cells generated with IL-4 and GM-CSF (Watanabe et al. 2009). These findings support the possible utility of *REIC/Dkk*-*3* gene therapy for not only breast cancers but also a broad range of human malignancies. Indeed, *REIC/Dkk*-*3* gene therapy is ongoing for prostate cancer (http://clinicaltrials.gov/ct2/show/NCT01197209).

Regarding the clinicopathological factors, we found that ER-positive breast cancer was more common in the methylated group than in the non-methylated group in the present study. In contrast, Veeck et al. (2009) reported that there was no correlation between *REIC/Dkk*-*3* methylation and ER and PgR statuses. In other cancers, no significant relationship was observed between the *REIC/Dkk*-*3* methylation status and any of the clinicopathological factors that were examined. Previous study has shown that *REIC/Dkk*-*3* methylation was associated with poor survival in primary breast cancers (Veeck et al. 2009). We could not compare these results directly because they examined the *REIC/Dkk*-*3* type-b methylation, which has a lower promoter activity than that of *REIC/Dkk*-*3* type-a. In addition, the number of cases in this study may have been too small for the survival analysis, since the primary purpose of this study was to detect *REIC/Dkk*-*3* type-a methylation and to examine the correlation between methylation and expression. In lung cancer, reduced expression of *REIC/Dkk*-*3* was previously shown to be frequent in poorly differentiated adenocarcinoma and squamous cell carcinoma (Nozaki et al. 2001). Further investigations are needed to determine the clinicopathological impact of *REIC/Dkk*-*3* type-a methylation.

In conclusion, we found that the promoter region of *REIC/Dkk*-*3* type-a was frequently methylated in breast, lung, gastric, colon, and prostate cancers and MPMs. *REIC/Dkk*-*3* type-a methylation and *REIC/Dkk*-*3* type-a mRNA expression were inversely correlated in the cell lines that were examined. Our results suggest that *REIC/Dkk*-*3* type-a methylation is an important mechanism in the pathogenesis of various types of malignancies. Since gene therapy using REIC/Dkk-3 expressing adenovirus vectors is currently ongoing for the treatment of prostate cancer, similar therapeutic modalities may be applicable for other types of cancers.
